# Expandable Mg-based Helical Stent Assessment using Static, Dynamic, and Porcine *Ex Vivo* Models

**DOI:** 10.1038/s41598-017-01214-4

**Published:** 2017-04-26

**Authors:** Youngmi Koo, Tarannum Tiasha, Vesselin N. Shanov, Yeoheung Yun

**Affiliations:** 1 NSF-Engineering Research Center, North Carolina A&T State University, Greensboro, NC 27411 USA; 2 FIT BEST Laboratory, Department of Chemical, Biological, and Bio Engineering, North Carolina A&T State University, Greensboro, NC 27411 USA; 3 Department of Chemical and Materials Engineering, University of Cincinnati, Cincinnati, OH 45221 USA

## Abstract

A bioresorbable metallic helical stent was explored as a new device opportunity (magnesium scaffold), which can be absorbed by the body without leaving a trace and simultaneously allowing restoration of vasoreactivity with the potential for vessel remodeling. In this study, developed Mg-based helical stent was inserted and expanded in vessels with subsequent degradation in various environments including static, dynamic, and porcine *ex vivo* models. By assessing stent degradation in three different environments, we observed: (1) stress- and flow-induced degradation; (2) a high degradation rate in the dynamic reactor; (3) production of intermediate products (MgO/Mg(OH)_2_ and Ca/P) during degradation; and (4) intermediate micro-gas pocket formation in the neighboring tissue *ex vivo* model. Overall, the expandable Mg-based helical stent employed as a scaffold performed well, with expansion rate (>100%) in porcine *ex vivo* model.

## Introduction

Atherosclerosis is a major cause of heart attack in the United States. Every year, approximately 700,000 Americans undergo percutaneous coronary stenting to alleviate coronary artery stenosis, at an estimated cost of $14 billion^1^. Durable metal stents, either bare metal or coated for “drug elution”, are usually fabricated from 316 L stainless steel (316 L), nickel-titanium (Nitinol), platinum chromium or cobalt-chromium alloy. These provide mechanical strength and generate large and stable lumens^2^. However, they are associated with in-stent restenosis and reocclusion rates, leading to long-term endothelial dysfunction, late thrombosis, toxic metal ion release, and local chronic inflammation^3, 4^. Antiplatelet therapy can mitigate many of these problems but increases the risk of bleeding. Polymer-based biodegradable stents address several of these problems^5–7^, but bring their own specific challenges: (1) they need to be expanded slowly; (2) struts need to be thick to provide sufficient mechanical strength, thus result in increased delivery system crossing profile, local flow disturbance predisposing to thrombosis, delayed endothelialization and increased restenosis; (3) polymeric scaffold may have a small increase in late thrombosis compared with second generation drug eluting stent (DES); (4) polymer degradation products can trigger inflammation and further thrombosis; (5) they are susceptible to fracture^8^.

Bioresorbable metal stents may offer significant advantages over current stent technologies^9, 10^ by providing solid scaffolding, safe degradation, and antiproliferative drug coating with no further late lumen loss related to neointimal growth^11^. They also secure structural support for a sufficient period of time before the stent degrades, which can mitigate the risk of causing a new disease (e.g. late thrombosis, in-stent restenosis, or neoatherosclerosis), and allow subsequent restenting at the same location if necessary^12^. Typical fabrication process of a bioresorbable metal stent involves^5, 13, 14^; (1) casting of metallic ingot, (2) thermomechanical treatment, (3) minitube drawing, (4) laser cutting, (5) annealing and acid pickling, and (6) electro-polishing. However, this process is complex and it can increase fabrication costs. Given the Mg-based metals’ mechanical properties for pure magnesium: (density of 1.74 gcm^−3^; elastic modulus of 45 GPa; compressive yield strength (0.2% CYS); ultimate compressive strength of 87 ± 4 MPa and 240 ± 9 MPa; tensile yield strength (0.2% YS) and ultimate tensile strength of 125 ± 9 MPa and 169 ± 11 MPa^15–17^), the conventional manufacturing approach of laser engraved Mg tubbing suffers from considerable limitations. The intrinsic low ductility of bioresorbable metals, which need to balloon-expand without losing mechanical strength, brings another limitation^17^. When a stent is expanded in the blood vessel, it is subjected to residual stress, cyclic loading from vessel contraction/expansion, and shear stress by blood flow^18, 19^. Despite reported clinical success^20^, the development of this potentially important device is still limited by a lack of detailed knowledge concerning the dynamic interaction between the degrading metal surface and the surrounding blood vessel’s physiology. As Mg scaffold degrades, it can possibly generate intermediate product, Ca/P complex deposition, particulates, gas pocket, which continuously interact with surrounding vessel, influencing cell viability, endothelialization, restenosis^21–24^.

Previously, the helically designed spiral stent demonstrated excellent flexibility and kink resistance, which allows uniform cell size at flexion points without scaling^25^. A helical stent with specific 3D geometric curvature will be patency-protective through stimulation of swirling flow and elevation of wall shear stress^26^. This study, for the first time, explores a bioresorbable metallic helical stent, which can potentially address current issues including expandability and manufacturing limitations, which can impede the mechanical performance and vessel and stent kinking, deformation and subsequent vessel trauma during flexion. The helical stent is fabricated by a patent-pending photochemical etching method, starting from 2D Mg-based ribbon coiled into a helical stent structure^27^. We report the systematic evaluation of a Mg-based helical stent, using *in vitro* and *ex vivo* models in terms of expandability, degradation behavior, and local tissue-stent interaction.

## Methods

### Helical stent fabrication

**Figure 1:**
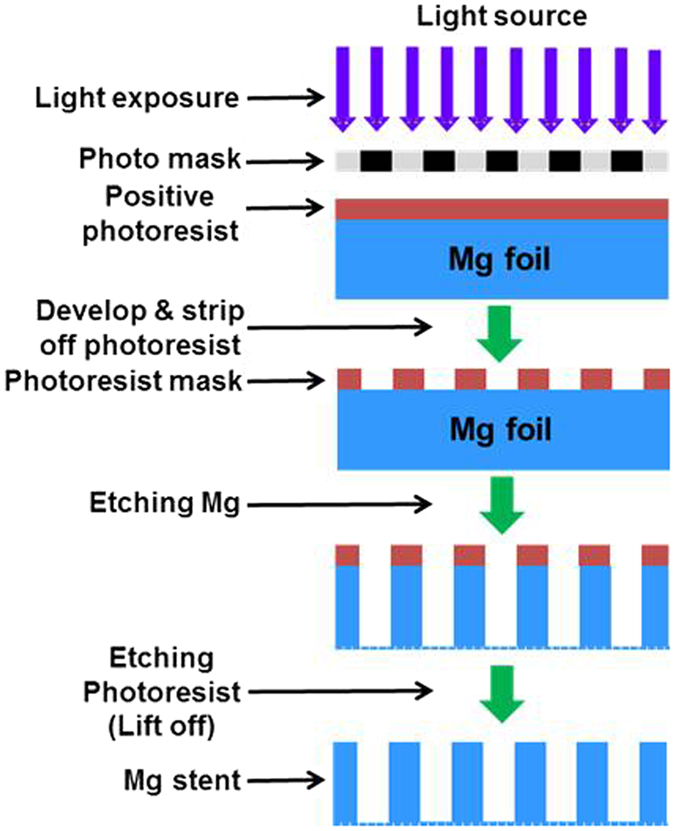
Schematic illustrating the manufacturing steps in the photo-chemical etching approach for making Mg helical stents.

The starting material is rectangular sheet of AZ31 magnesium alloy (94% Mg, 3% Al, 1% Zn) with dimensions 200 mm × 500 mm and thickness of 250 μm, purchased from Goodfellow, Oakdale, PA. We have developed a novel approach to manufacture stents - photochemical etching (Fig. 1), which is simple and has good accommodation for design changes and high throughput. This method involves photolithographically transferring a stent texture on the foil, followed by chemical etching. The photochemical etching generates no residual stress in the material during the manufacturing process. This patent-pending process for stent fabrication is simple, affordable and fast. Further, it is scalable and can produce hundreds of stents at once on a 4 ft × 6 ft Mg sheet. Finally, no post-manufacture treatment of the stent, such as welding, annealing or etching, is required.

### Artery sampling for *ex vivo* testing

Porcine abdominal aortas were obtained from Piedmont Custom Meats, Inc. (9683 Kerr Chapel Road Gibsonville, NC, USA 27249) operating under the North Carolina Meat and Poultry Inspection Division. This permission and all procedures were carried out in strict accordance with the regulation of the North Carolina Department of Agriculture and Consumer Services (NCDA&CS, Meat and Poultry Inspection Division, USA). All protocols were approved by North Carolina A&T State University Animal Care and Use Committee (IACUC), and performed according to IACUC guidelines. Each excised artery was immediately rinsed with ice-cold phosphate buffered saline (PBS) solution, then stored in the cold simulated body fluid (SBF) solution consisting of the Dulbeco’s modified Eagle’s medium (DMEM) with 10% fetal bovine serum (FBS) and 1% penicillin-streptomycin (P/S) on ice.

### Bioreactor

**Figure 2:**
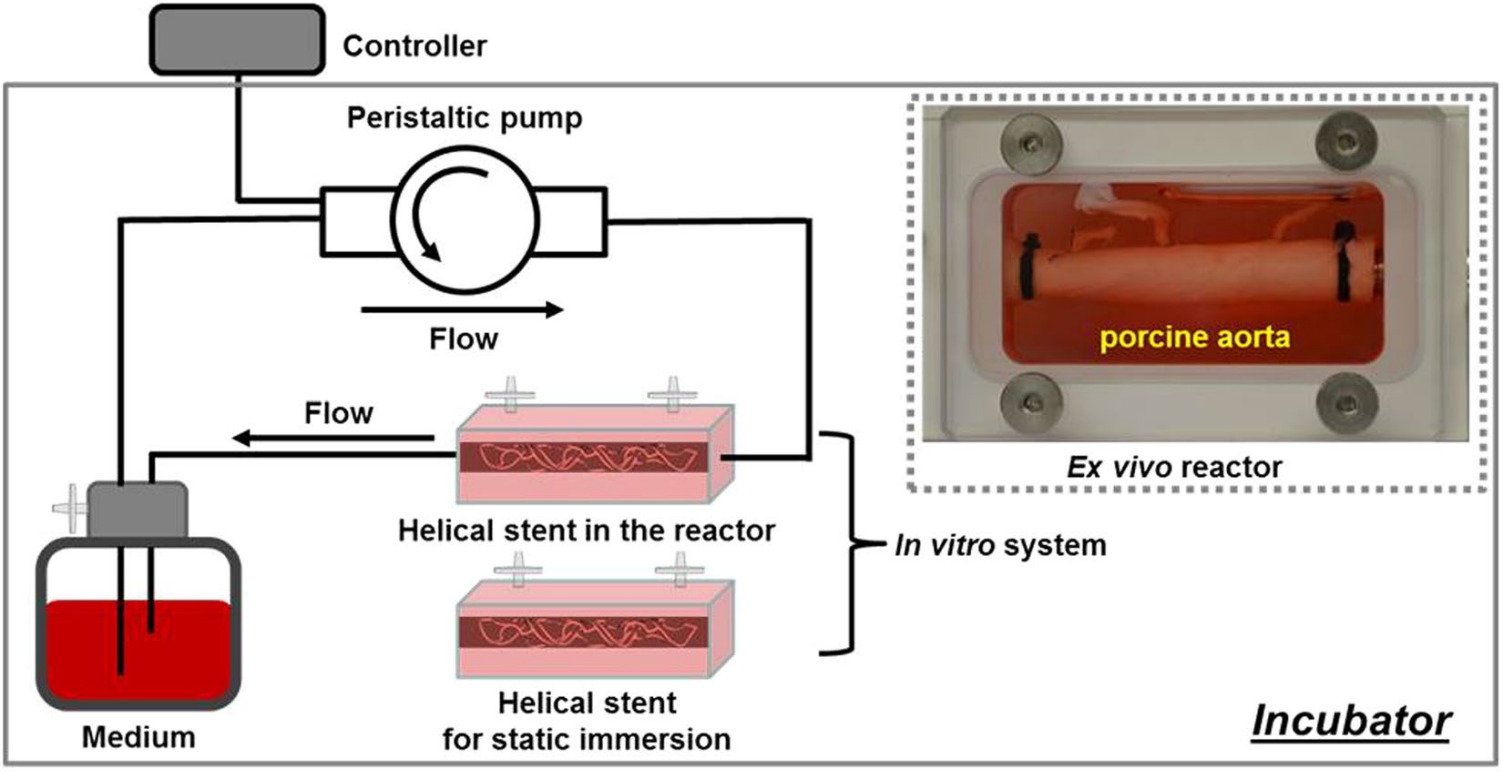
Bioreactor configuration for the *in vitro* and *ex vivo* simulation of the Mg-based helical stent.

A bioreactor was used to provide an *in vitro* dynamic flow environment for the Mg-based helical stents (Fig. 2). The apparatus consisted of: (1) a reactor: (2) a flow transfer pump: (3) a reservoir: and (4) a flow controller. All flow channels were autoclaved before use. Helical stents were deployed in silicone tubing (EW-95802-04, EW-95802-07, Cole-Parmer, IL, USA) for dynamic *in vitro* modeling or in porcine artery for *ex vivo* modeling. Helical stents were inserted on a balloon catheter into artificial vessel or arteries with 10 cm length (Table 1). The balloon expansion was deployed against the vessel wall. Both ends of the stented vessels were connected with nylon cable ties to the bioreactor and the simulated body solution was circulated. Fluid dynamics test was performed in a humidified incubator maintained at 37 °C in 5% CO_2_. Static immersion was simultaneously carried out under same environmental condition.

**Table 1:** Experimental conditions for *in vitro* artificial vessel and *ex-vivo* artery.

Method	Vessel diameter (mm)	Wall thickness (mm)	Max. Expandable rate (%)	Flow rate (mL/min, when 0.68 Pa)
Static	3.175	1.8	—	—
Dynamic	3.175	1.8	0	136.3
	3.175	1.8	~58.8	136.3
	3.969	1.6	~98.5	266.3
Porcine *Ex vivo*	8.5	2.5	~112.5	592.4*

^*^Flow rate (mL/min) was operated according to the 0.154 Pa.

### Dynamic flow simulation

The dynamic flow applied on inner surface of the helical stent was controlled by modifying the flow rate of solution according to the following formula^28^.

1 $${\rm{\tau }}=32{\rm{\eta }}Q/({{\rm{\pi }}{\rm{D}}}^{3})$$

where, τ and Q are the shear stress and laminar flow rate, respectively. η is the shear viscosity of the fluid, the dynamic flow viscosity of DMEM with 10% FBS at 37 °C is 0.94 mPa·s^29^. D is vessel diameter.

Table 1 summarized the diameter of the lumen in artificial vessels and arteries. For *in vitro* vessel reactor, we applied mean wall shear stress of coronary artery, 0.68 ± 0.03 Pa^28^. *Ex vivo* artery reactor was operated according to the intrarenal aorta^30^. Expansion was defined as follows;

2 $$Expansion\, \% =\frac{{R}_{Final}-{R}_{Initial}}{{R}_{Initial}}\,\times 100$$

where, *R* is the inner diameter of the stent.

### Micro X-ray computed tomography imaging

3D images of all the Mg-based helical stents before/after expanding were examined using X-ray computed tomography (CT, GC Phoenix Nanotom-M^TM^, GE Sensing & Inspection Technologies GmbH). We applied 100 kV voltage and 90 μA current and all images were reconstructed using phoenix datos | x software provided with the respective micro-CT systems. Moreover, degradation rates (mm/year) of the Mg-based helical stents were calculated from the volume ratios and surface areas determined by micro-CT. The modified equation for degradation rate as follow was used^31^.

3 $${\rm{DR}}=\frac{{\rm{\Delta }}V}{At}$$

where, DR is the degradation rate, *∆V* (mm^3^) is the reduction in volume that is equal to the remaining Mg-based helical stent volume after removing degradation product, *A* (mm^2^) is the surface area of the pristine Mg-based helical stent exposed to the simulated body fluid and *t* (year) is the exposure time.

### Histology

Porcine aorta (artery) samples before (only artery) and after (artery with stent) *ex vivo* testing were fixed in 4% paraformaldehyde at 4 °C for 24 hours. Samples were dehydrated through an ethanol gradient of 80%, 90% for 30 mins twice, and 100% for 1 hour twice at 4 °C. To remove lipids from the tissue for facilitating penetration of the embedding medium before embedding in Technovit^®^ 9100, the samples were defatted in xylene for 1 hour twice at 4 °C. The samples were embedded in a low-temperature embedding system, Technovit^®^ 9100 (Heraeus Kulzer GmbH, Germany). Infiltration and polymerization solutions were prepared according to manufacturers’ instructions, and arties were pre-infiltrated, infiltrated at 4 °C and polymerized at −2 °C for 5 days. 10 μm-thick sections (TC-65 tungsten carbide blade, RM2255 Rotary Microtome, Leica Biosystems of Technovit^®^ 9100) were stretched with 70% ethanol on Superfrost Plus slides (Fisher Scientific, USA) and dried overnight at 50 °C. Technovit^®^ 9100 sections were immersed in 2-methoxyethyl acetate (MEA) for 1 × 20 minutes followed by xylene for 2 × 20 minutes, and then in high-purity acetone for 4 × 5 minutes. The samples were evaluated histologically with hematoxylin and eosin (H & E) stain. Image acquisition was carried out with a light microscope (Olympus IX 83, USA).

### Structural morphology and elemental analysis

The surface morphology of the helical stents (*in vitro*-tested and Technovit^®^ 9100 embedded/sectioned after *ex vivo* testing) was examined by FE-SEM (Hitachi 8000), operating at 5 kV. The samples were gold-coated for FE-SEM measurements. EDX data were obtained with a Bruker AXS (XFlash detector 5030) attachment on the FE-SEM at 10 kV accelerating voltage and 15 mm working distance.

### Statistical analysis

Data were analyzed by one-way ANOVA followed by comparing the values of corresponding Markers. A level of P < 0.05 was considered statistically significant. All results were presented with the mean ± standard deviation (SD).

## Results

### Mg-based helical stent for *in vitro* and *ex vivo*

The fabricated Mg helical stent is designed to be coiled easily by hand on a guiding cylinder. Its shape can be manipulated and molded before or after balloon delivery in the vessel. The thickness of the stent wire was designed for 250 μm to meet radial stress requirements (Fig. S1a,b). The Mg-based helical stent show uniformly radial enlargement during the balloon expansion (Fig. S1c). The helical shape allows creating stent geometry that promotes swirling of intraluminal blood flow.

**Figure 3:**
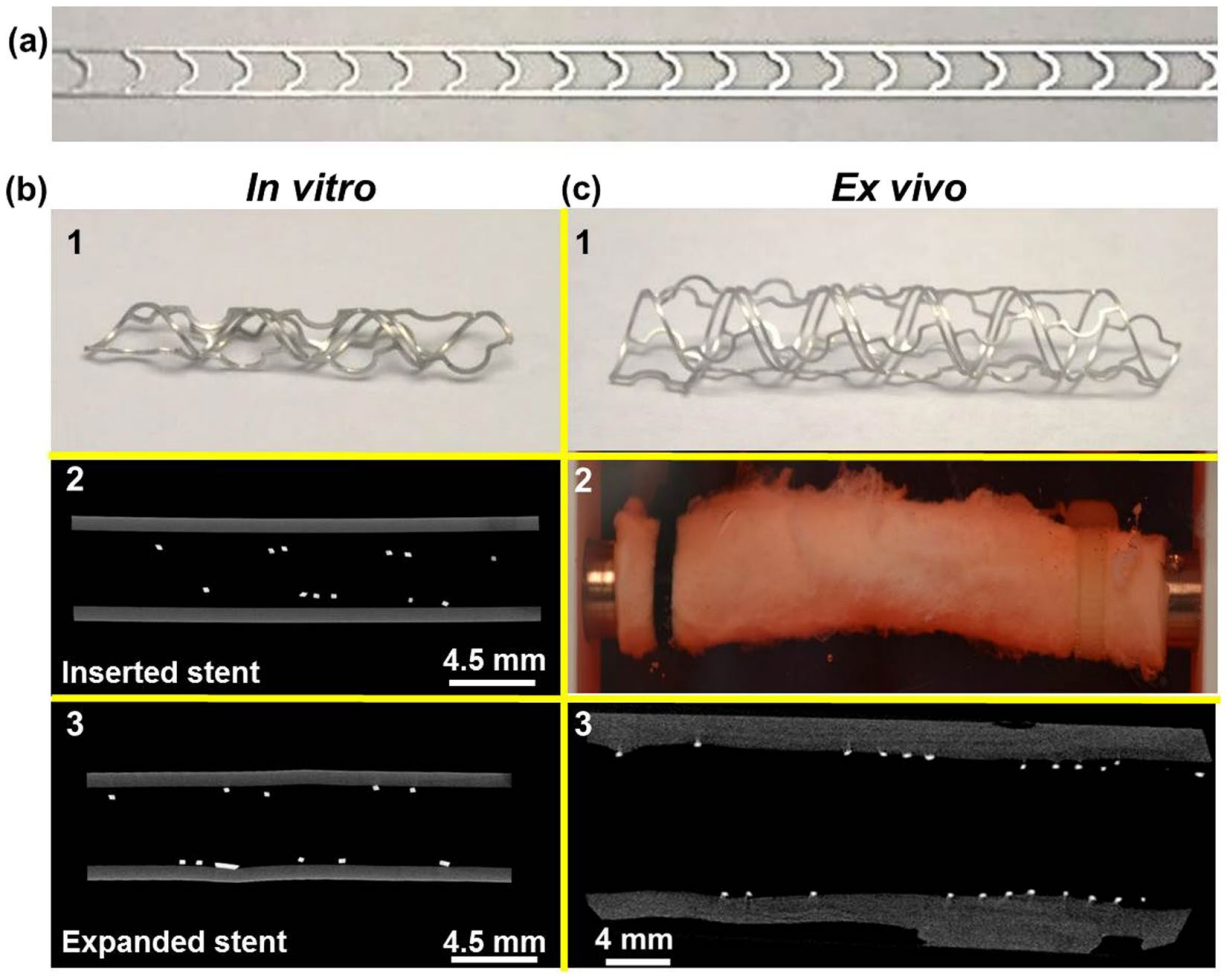
Geometries and stenting of Mg-based helical stent for vascular bioreactor tests. (**a**) Optical image of 2D Mg-based ribbon before coiling to form the device, (**b**) *In vitro* stent testing: optical image of helical stent for *in vitro* test (b1), inserted stent in artificial vessel (b2), and expanded stent in vessel (b3), (**c**) *Ex vivo* stent testing: optical image of helical stent for *ex vivo* (c1), optical image of the inserted stent in porcine artery (c2), and expanded stent in artery (c3).

Figure 3a shows a 2D ribbon, which was shaped to form Mg-based helical stents with two different sizes. These two devices with different dimensions were deployed/expended in artificial vessel for *in vitro* (Fig. 3b) and porcine artery for *ex vivo* (Fig. 3c) based on the Table 1 condition. Figure 3b shows a Mg-based helical stent (2 mm dia. × 20 mm length) deployed/expanded to ~98.5% in artificial vessel for *in vitro* test. Figure 3c displays helical stents for *ex vivo* bioreactor test fabricated with 4 mm dia. × 30 mm length and deployed/expanded in the porcine artery. The vessels with different artificial diameters, (3.175~3.969 mm *in vitro*), were used to study expandability for: 1) 0%; 2) ~58.8%; and 3) ~98.5% respectively.

### *In vitro* bioreactor

**Figure 4:**
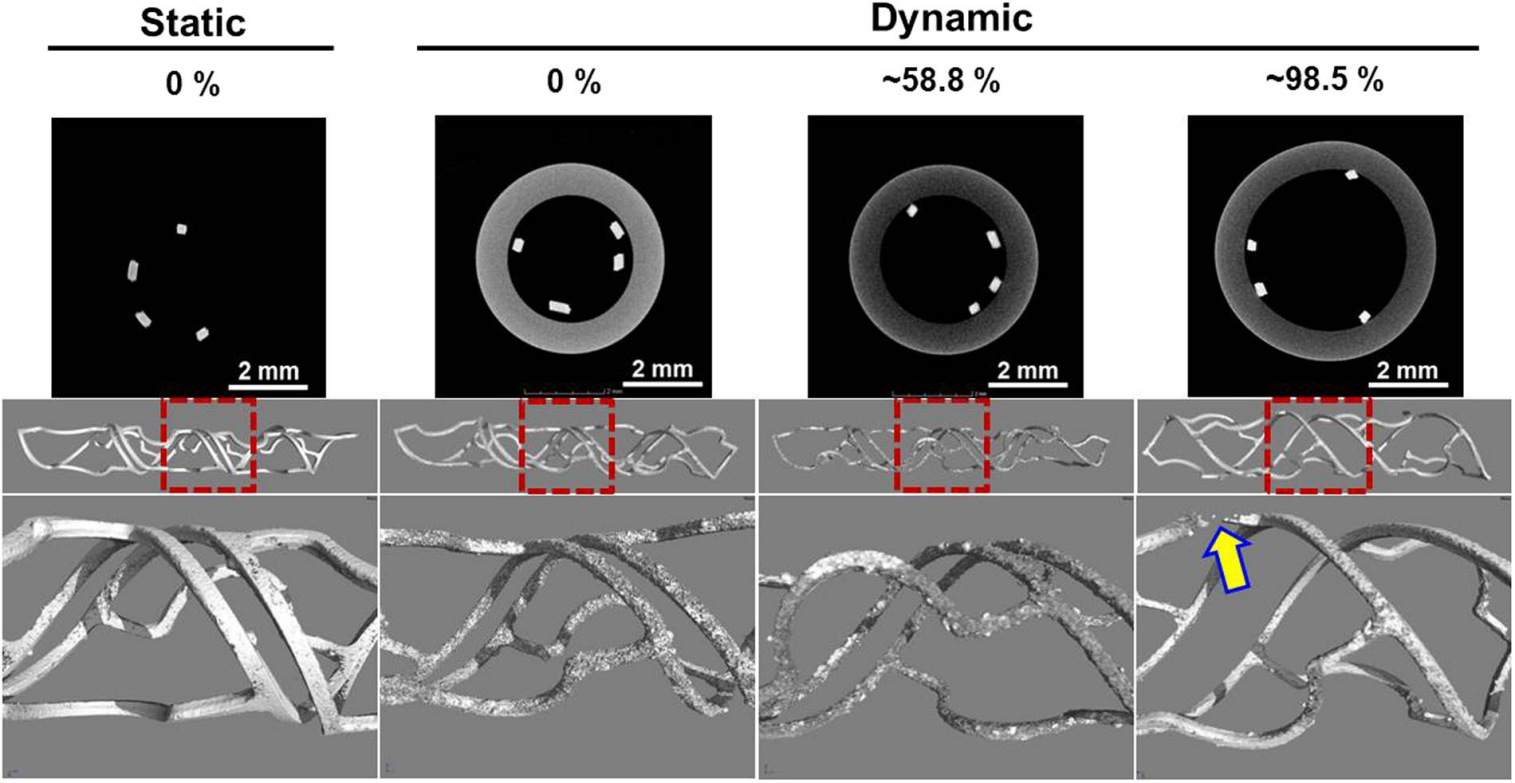
Cross-sectional 2D (before test) and 3D (after test) micro-CT structure images revealing corrosion products accumulated during the *in vitro* static and dynamic fluid flow simulations for 3 days in DMEM (10% FBS, 1% P/S) at 37 °C, 5% CO_2_. Red dot box images were enlarged. Yellow arrow indicates broken strut after *in vitro* dynamic fluid flow simulation.

Figure 4 shows micro-CT images before (2D cross-section image) and after stent degradation from (1) static immersion and (2) dynamic bioreactor (wall shear stress, 0.68 Pa) undergoing expansion rates of 0%, ~58.8%, and ~98.5%, after 3 day exposure. Stent degradation from static immersion shows that the surface of helical stent appeared relatively smooth, indicating a lower degradation rate. On the other hand, the surface of helical stent from the dynamic test had more coverage with a degradation product layer, suggesting a higher degradation rate. In particular, we observed a broken strut in the 98.5%-expanded stent (yellow arrow in Fig. 4) during degradation process. Residual stent volume (Fig. S2) of all helical stents before/after removing degradation products under static immersion has the slowest degradation rate (17.57% ± 1.89% volume loss, 3 days), whereas the helical stent (0% expansion) in dynamic bioreactor was rapidly degraded (43.30 ± 4.89% volume loss). The two helical stents (~58.8% and ~98.5%) which expanded within the artificial vessel and tested under dynamic reactor show similar degradation rates regardless of expansion rates.

**Figure 5:**
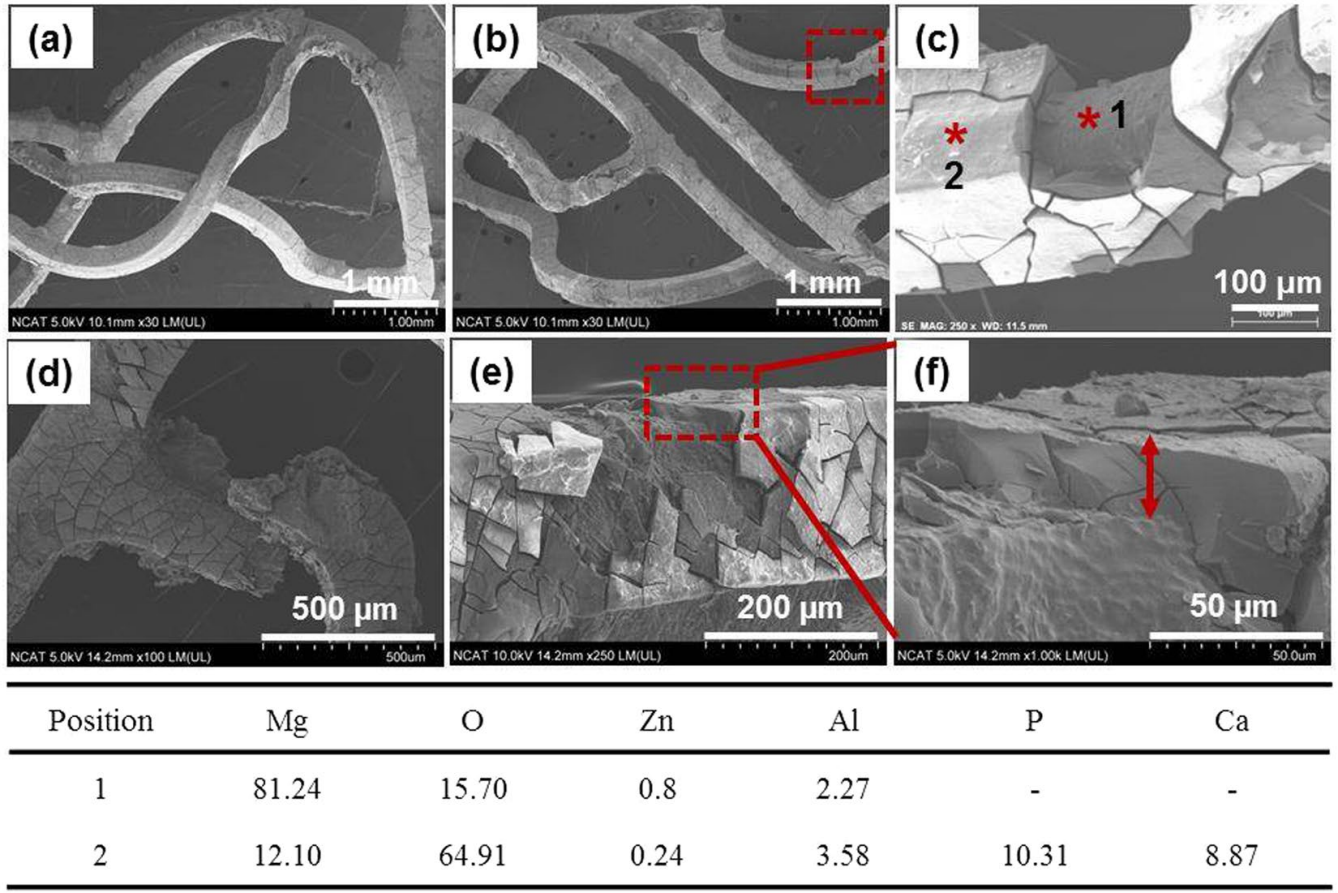
SEM images of degraded areas on tested helical stents under flow induced shear stress, 0.68 Pa for 3 days in DMEM (10% FBS, 1% P/S) at 37 °C, 5% CO_2_. (**a**–**c**) Represent the ~58.8% expanded helical stent, (**d**–**f**) represent the ~98.5% expanded helical stent. The Table displays the atomic percent (at %) detected in the two positions by EDX.

Figure 5 shows degradation products and morphologies of two differently expanded helical stents, analyzed by SEM and EDX. The surfaces of the stents were mainly covered by degradation products, which were partly detached (Fig. 5b,c of ~58.8% expansion and Fig. 5e,f of ~98.5% expansion). The ~98.5%-expended helical stent images (Fig. 5d) also show a broken strut and a corroded strut surrounded by degradation product. The thickness of degradation product was about 25 µm in a flat region and 40 µm in an edge region of the strut. EDX data in Fig. 5 shows the chemical composition with each position of the ~58.8% expanded helical stent in Fig. 5c. The predominant elements of the inner side (position 1) were Mg and O. We detected Al and Zn on the surface, of which AZ31 contains 3% and 1%. Compared with position 1, the surface of degraded helical stent (position 2) showed significant amount of P and Ca along with the same elements detected in position 1 (Mg, O, Zn, and Al).

### Porcine *ex vivo* model and stent-arterial tissue interaction

**Figure 6:**
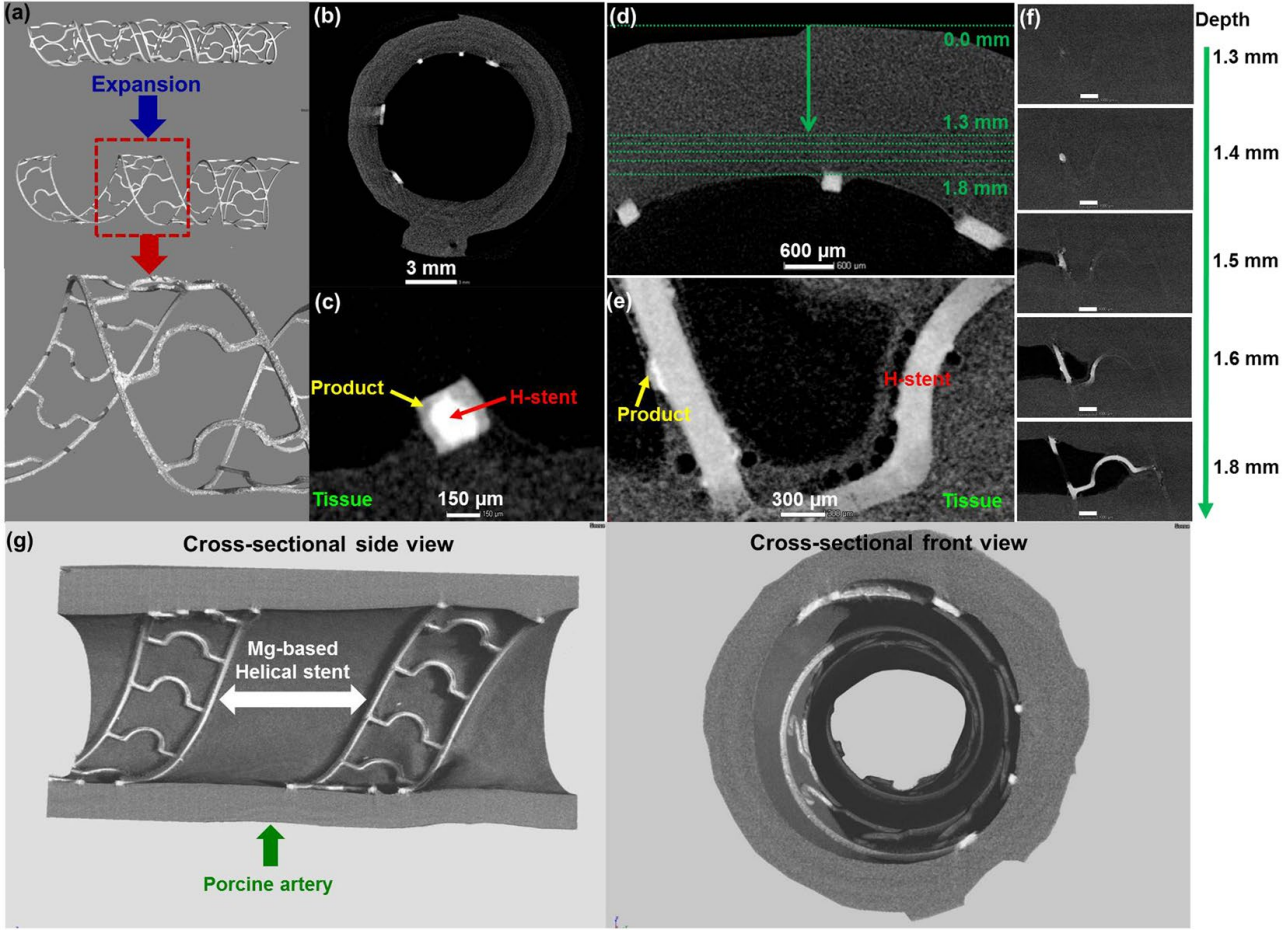
3D structure and cross-sectional 2D micro-CT images of expended helical stents in artery revealing the with corrosion products *ex vivo* dynamic simulation for 3 days in DMEM (10% FBS, 1% P/S) at 37 °C, 5% CO_2_. (**a**) Bare stent and extracted stent in artery, (**b**) 2D sliced image, (**c**) Enlarged one strut of (**b**), (**d**) Interface between implanted helical stent and tissue, (**e**) Enlarged images at a depth of 1.6 mm in (**d**,**f**) sliced images from outmost of artery to lumen (arrow direction in (**d**)), (**g**) Actual representative X-ray micro-CT 3D structures of expanded Mg-based helical stent scaffold at the porcine artery. Product: Ca/P complex, H-stent: Mg helical stent, Scale bars on (**f**): 1 mm.

Figure 6 shows *ex vivo* testing results of helical stents (calculated expansion rate, ~112.5%), which were implanted in the artery under wall shear stress of 0.154 Pa for 3 days. Figure 6a column shows 3D micro-CT image: (1) bare helical stent; (2) expanded helical stent and extracted from artery with degradation product; and (3) enlarged view of extracted helical stent. Figure 6b displays a representative 2D cross-sectional image of helical stent with arterial tissue. Figure 6c shows the degradation process of the helical stent including: (1) helical stent by itself (white color) and (2) product portion (gray color), retaining its original shape. Helical stent was well in-stented in artery vessel. In Fig. 6d–f, the Mg-based helical stent was deployed with the flap intima tissue, which was degraded and surrounded by local tissue. Despite the formed degradation products the stent remained intact^32^.

Figure 6f shows the sliced-images with depth of helical stent-artery in Fig. 6d. Figure 6e shows a stent-tissue interaction image from outermost of artery to lumen, retaining intermediate microbubbles between the stent and local tissue. We also observed localized crystalline deposits (yellow arrow) on the stent’s surface. This primary study demonstrated that the helical Mg scaffold was in-stented in vessel and degraded with expected time period. Figure 6g shows actual representative X-ray micro-CT 3D structures of expanded Mg-based helical stent in the porcine artery of cross-sectional side view (left) and cross-sectional front view (right). The helical Mg scaffold was largely expanded (>100%), in-segmented, and well-apposed (in contact with the vessel wall). Reconstructed CT image provide perspective the spiral extending into the plane of the image as seen in the right hand image.

**Figure 7:**
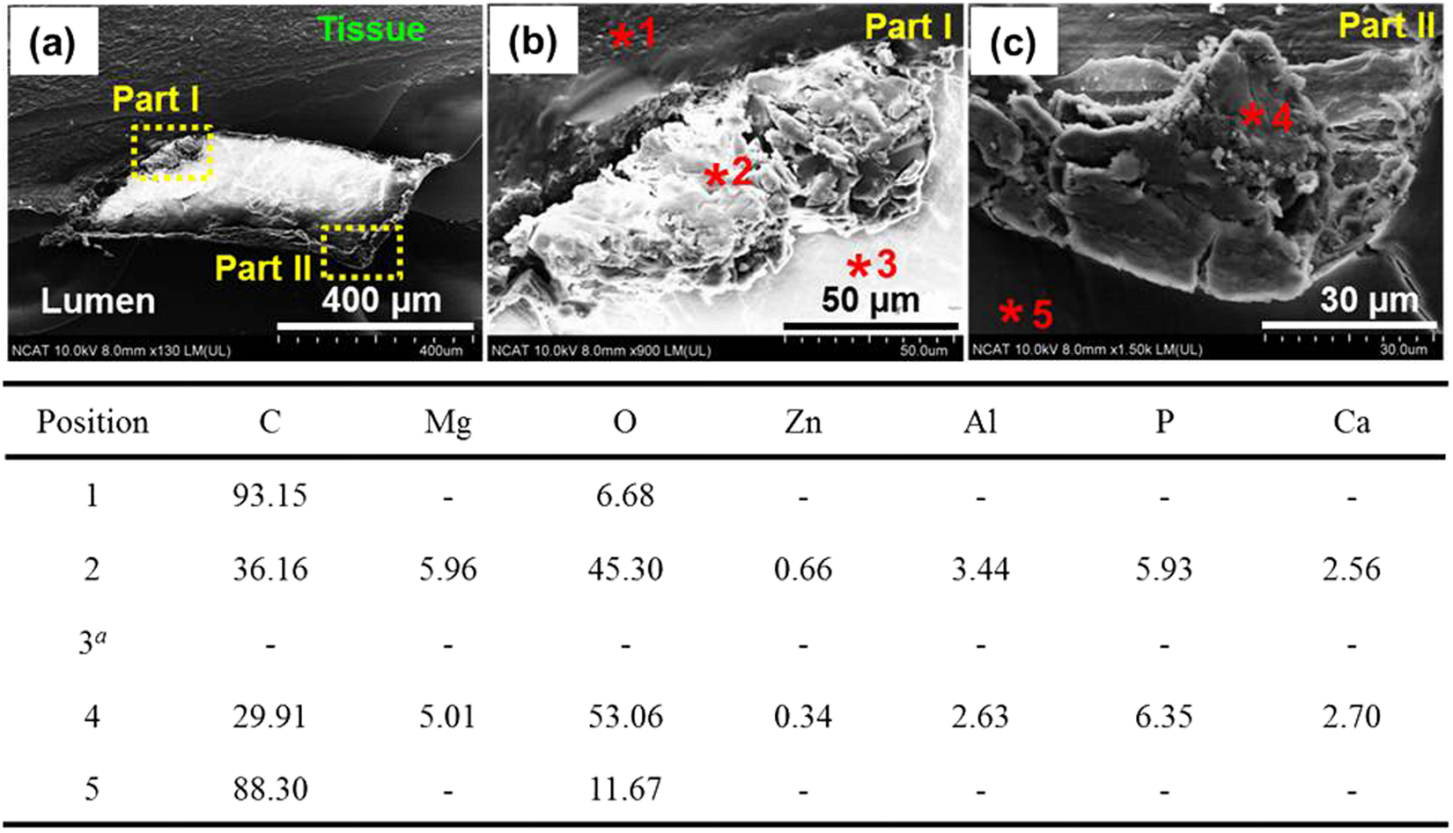
SEM images and EDX analysis of corrosion products surrounding expanded helical stents under the flow induced wall shear stress value of 0.154 Pa for 3 days in DMEM (10% FBS, 1% P/S) at 37 °C, 5% CO_2_. ^*a*^Indicates Mg-based helical stent has been implanted.

We embedded helical stent-tissue artery into Technovit 9100 resin and sectioned it with 10 μm thickness for studying the interface between implanted stent and artery. Figure 7 shows SEM and EDX images for the chemical composition after 3 days dynamic *ex vivo* test. Even though there were residual stent pieces in arterial tissue, they disappeared during the sample preparation. However, we still observed degradation product which tissue has remained intact (Fig. 7a). We further analyzed the chemical elements in the degradation region of helical stents (two parts): (1) the product located near to artery (Fig. 7b: part I of Fig. 7a), and (2) the product located near to the lumen (Fig. 7c: part II of Fig. 7a). The observed elements on the helical stent-implanted tissue largely depended on the analysis position. EDX results show that tissue (position 1) and lumen (position 5) mainly consisted of C and O (>88% and >6%), mainly from embedding resin material. In position 3, the stent strut was presented until embedding process; C and O were observed, but they were ignored since their peak intensity was too low compared with the signal-to-noise ratio. The main elements of degradation products that existed in both edges (adjacent tissue and lumen) were C, O, P, Ca, and Mg, as well as trace amounts of Mg alloying elements (Zn and Al). Even though we could not show histological result due to the position of the stent scaffold exposed very close to lumen, adjacent artery on strut after *ex vivo* test confirmed the health regarding the structure of the extracellular matrix, as shown in H & E image (Fig. S3).

## Discussion

Ideal stent needs well-suited mechanical properties such as a high elastic modulus to prevent stent recoil and low yield strength to allow stent expansion^33^. In this study, we did not observe distinctive recoil of helical Mg scaffold in vessel after large expansion (Fig. 6g), which may be one of the good advantages to address current challenges of Mg-based stents. This helical stent can possibly match performance of radial elastic recoil and flexibility of commercially available stents^34, 35^.

In the degradation analysis of bioresorbable Mg-based stent scaffold, degradation rates of the stents under the static immersion, and *in vitro* flow environments were compared (Table 2). The stent in static immersion was degraded slower than that the stent under *in vitro* dynamic artificial vessel bioreactor. We found that flow induced wall shear stress *in vitro* vascular bioreactor is a significant factor affecting degradation. The 0%-expanded stent within the artificial vessel (malapposed) has the highest degradation rate due to the exposed device surface to the dynamic flow rate. We observed that fully expanded helical stents (~58.8% and ~98.5%) with each artificial vessel lumen size (Table 1) had a similar degradation rate (1.62 ± 0.10 and 1.64 ± 0.23 mm/year) regardless of the degree of expansion. However, the stent scaffold of the larger expansion (98%) was affected by the degradation rate, causing strut fracture (Fig. 4) due to mass transfer and mechanical load^36, 37^. The degradation rate of the bioresorbable metallic helical stent was more affected by turbulent tubular flow at the stenting location (Fig. 4).

**Table 2:** Average degradation rates of Mg-based helical stent under *in vitro*.

	Static immersion	Dynamic artificial vascular bioreactor*		
Expansion rate (%)	0	0	~58.8	~98.5
Degradation rate (mm/y)	0.99 ± 0.20	2.44 ± 0.45	1.62 ± 0.17	1.64 ± 0.39

^*^Flow rate (mL/min) was operated according to the wall shear stress of 0.68 Pa.

Table 3 shows summary of bioresorbable metallic stent degradation rates in terms of alloys, design, methods and wall shear stress. Our results suggest 0.44 mm/year (compete degradation time, 6–8 months based on the assumption that stent scaffold degrades uniformly) and others have reported similar range from 0.37 to 1.21 mm/year^19, 37, 38^. One clinical study reported 12 months for complete degradation (polymer-coated Mg scaffold) and explored more in-segment late lumen loss (0.27 mm to 0.36 mm) and revascularization at 6 months^11^. A study using an *ex vivo* model demonstrated a degradation rate two times slower than that in static immersion *in vitro*, suggesting that scaffold-lumen interactions such as chemical interaction (degradation, protein deposition), mechanical interaction (fibrous encapsulation, calcification), surface interaction (protein adsorption, provisional matrix formation of SBF solution-stent) governed the rate of stent degradation^39^. Intermediate products of stent scaffold according to the physiological flow environment can be detached from host scaffold and degraded slowly. As shown Fig. 6c, intermediate products in *ex vivo* capsulated host stent scaffold during gradually resorption process. A recent article^11^ suggests two steps of Mg scaffold degradation: first, the magnesium alloy is converted to hydrated magnesium oxide, which is then is transformed to magnesium phosphate, which is consecutively replaced by amorphous calcium phosphate. During this process, metallic magnesium is removed by diffusion from the amorphous matrix and is absorbed by the body. The amorphous calcium phosphate remains in the tissue together with the other elements of the alloy and the markers^11^. We observed a similar degradation process, with intermediate products including hydrated MgO/Mg(OH)_2_, soft amorphous hydroxyapatite or Ca/P compound. For example, EDX element analysis of malapposed (protruding into the lumen at a distance greater than the strut thickness) helical stent after *ex vivo* test under dynamic flow for 3 days indicates degradation products and deposition of soft amorphous hydroxyapatite (Fig. S4).

**Table 3:** Comparison of the bioresorbable metallic stents.

Material	Strut material	Design	Model	Shear stress (Pa)	Testing period (day)	Method	Degradation rate (mm/y)	Literature
Mg-base	AZ31	Helical Coil	*Ex vivo* (SBF)	0.154	3	Volume reduction	0.44	This article
	AZ31	Helical Coil	Immersion (SBF)	—	3	Volume reduction	0.99	This article
Mg-base	AZ31	Tubular	Immersion (SBF)	—	7	Volume reduction	0.37	37
			Dynamic (SBF)	0.056	7	Volume reduction	1.21	
Mg-base	AZ31	Tubular	Immersion (D-Hanks’ solution)	—	3	Mass loss	23~35% mass loss	19
Fe-base	Fe35Mn	Prototype	Dynamic (SBF)	0.6		Electro chemical	0.51	38

A previous *in vivo* study in bioresorbable Mg-based alloys has reported on their high compatibility with body tissue and intrinsic dissolution in body fluids^40^. Early clinical studies of a balloon-expandable Mg alloy bioresorbable stent by Biotronik, poly-L-lactic acid (PLLA) self-expanding stent by Kyoto Medical Planning, as well as bioresorbable vascular scaffold (BVS) by Abbott have been reported^10, 41, 42^. In particular, the Mg stent was presented as well-expanded on deployment without immediate recoil or high restenosis rate with an in-stent late loss^43^. However, in ongoing efforts to develop a bioresorbable metallic stent scaffold, previous studies also reported several limitations: (1) fast resorption, (2) recoil of lower strength compared to metallic stent, (3) relatively poor crossing profile, and (4) radiolucent. Stent implantation can cause malapposition between the strut and the endothelium, leading to high wall shear stress and perturbation of the local flow field^44^. This perturbed flow can create a risk of thrombosis^45^. Systematic studies of: (1) static immersion, (2) *in vitro* intraluminal flow, and (3) *ex vivo* intraluminal flow environments demonstrated the potential use of bioresorbable metallic helical stent. This paper tries to provide proof-of-concept for innovation opportunity of bioresorbable metallic Mg scaffold stent. In future studies, we would optimize the design and pattern of the 2D bioresorbable metallic ribbon, spiral coiling number, strut thickness, crossing profile, device diameter, lower percentage of wall coverage, and achieve swirling laminar blood flow. Also, long term *in vivo* studies are needed to assess overall resorption time, late lumen loss (LLL), neointimal hyperplasia, vasomotion, and revascularization.

## Electronic supplementary material

Supporting Information
