# Supplementary material for: Expandable Mg-based Helical Stent Assessment using Static, Dynamic, and Porcine Ex Vivo Models
Source: Sci Rep. 2017 Apr 26;7:1173. doi: 10.1038/s41598-017-01214-4 (PMC5430820; doi:10.1038/s41598-017-01214-4)
Supplement: Supplementary file 1 — Supporting Information [file 41598_2017_1214_MOESM1_ESM.pdf]

Supporting Information

Expandable Mg-based Helical Stent Assessment using Static, Dynamic, and Porcine *Ex Vivo* Models

Youngmi Koo[a,b], Tarannum Tiasha[c], Vesselin N Shanov[c], and Yeoheung Yun[a,b*]

[a] NSF-Engineering Research Center, North Carolina A&T State University, Greensboro, NC 27411, USA

[b] FIT BEST Laboratory, Department of Chemical, Biological, and Bio Engineering, North Carolina A&T State University, Greensboro, NC 27411, USA

[c] Department of Chemical and Materials Engineering, University of Cincinnati, OH 45221, USA

S1. Mg-based helical stent used in this study

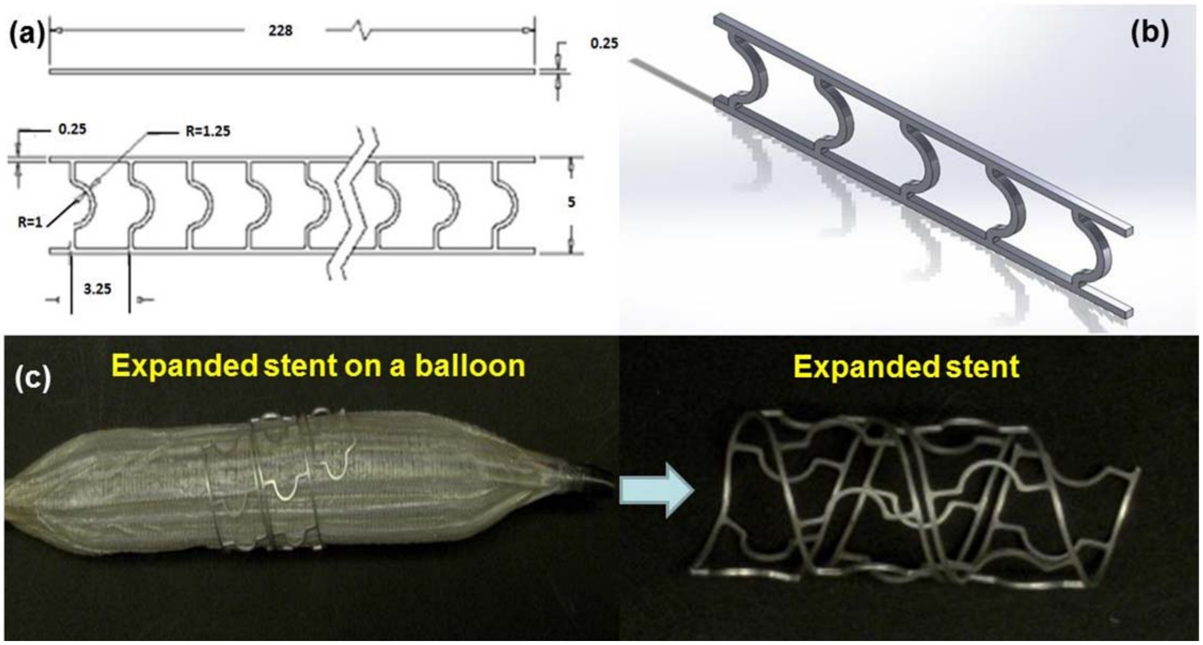

Fig. S1. (a) Design with dimensions in metric units, (b) 3D structure of (a), (c) Optical image of Mg-based helical stent with initial outer diameter 4 mm and length 20 mm sitting expanded on a balloon and expanded Mg helical stent.

S2. Residual stent volume after *in vitro* testing

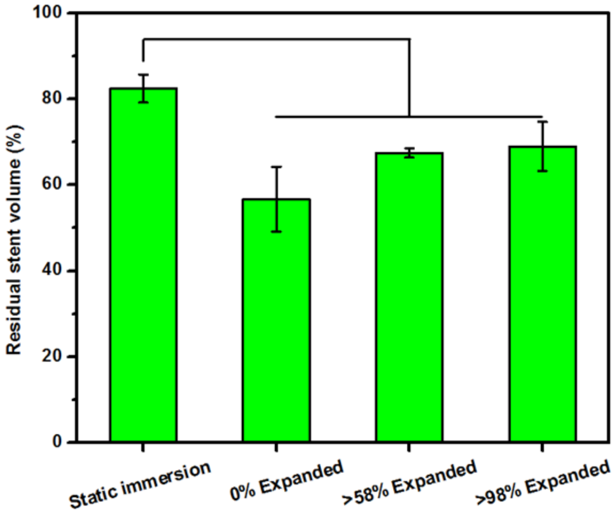

Fig. S2. Comparison of residual stent volume of helical stents after *in vitro* test for 3 days in DMEM (10 % FBS, 1 % P/S) at 37 ºC, 5 % CO_2. *$p < 0.005$.

S3. Histological analysis of the porcine aorta between strut and strut after *ex vivo* test

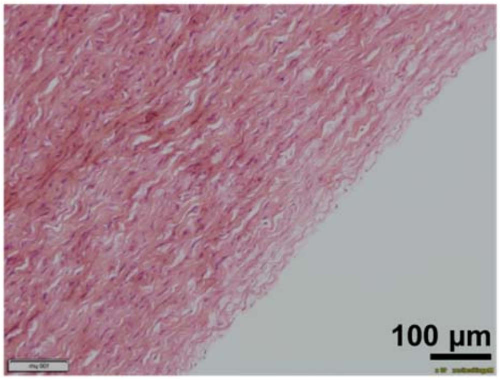

Fig. S3. H & E staining of adjacent artery on helical stent strut after *ex vivo* test for 3 days.

S4. EDX analysis of malapposed helical stent part in artery

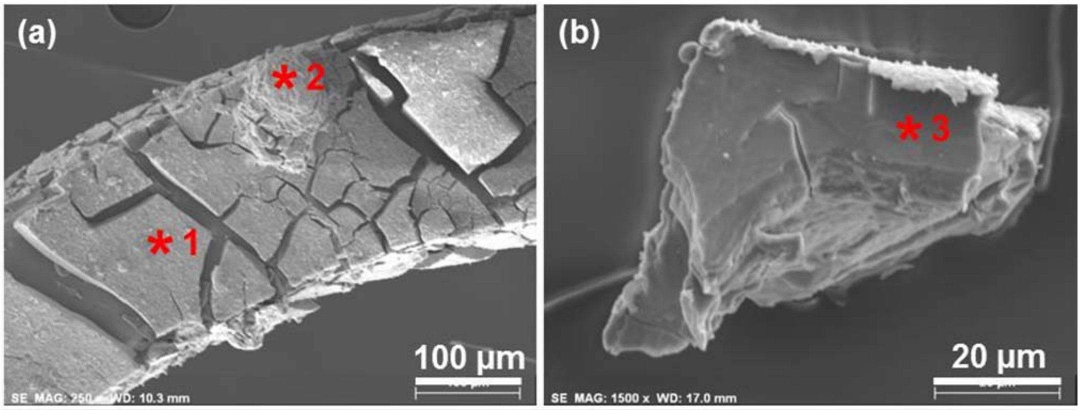

Position	C	Mg	O	Zn	Al	P	Ca
1	61.65	3.11	23.00	-	1.21	10.80	0.23
2	26.89	-	41.10	-	-	13.73	18.27
3	0.41	15.76	41.13	-	8.79	22.66	11.25

Fig. S4. EDX element analysis of malapposed helical stent part in artery with expanded helical stents after *ex vivo* test under flow induced shear stress values of 0.154 Pa for 3 days in DMEM (10 % FBS, 1 % P/S) at 37 ºC, 5 % CO_2. (a) Stent strut with degradation products, and (b) chunk of degradation product which was detached from stent strut after test.
